# Characterization of Two *Pseudomonas aeruginosa* Viruses vB_PaeM_SCUT-S1 and vB_PaeM_SCUT-S2

**DOI:** 10.3390/v11040318

**Published:** 2019-04-01

**Authors:** Yangyijun Guo, Ping Chen, Zhanglin Lin, Tingting Wang

**Affiliations:** School of Biology and Biological Engineering, South China University of Technology, 382 East Outer Loop Road, Higher Education Mega Centre, Guangzhou 510006, China; 201620133950@mail.scut.edu.cn (Y.G.); chenp211@scut.edu.cn (P.C.)

**Keywords:** *Pseudomonas aeruginosa*, phage therapy, *Myoviridae*, complete genome, proteomics, one-step growth curve, lysis kinetics, biofilm

## Abstract

The sophisticated antibiotic resistance mechanism of *Pseudomonas aeruginosa* has urged the development of alternative antibacterial strategies. Phage therapy has been proven successful for the treatment of multidrug-resistant infections. In this study, we reported two virulent *P. aeruginosa* phages, vB_PaeM_SCUT-S1 (S1) and vB_PaeM_SCUT-S2 (S2), which were characterized at morphological, genomic, and proteomic levels. Phages S1 and S2 were assigned to the *Myoviridae* family. The genome sequencing showed that the genome size of Phage S1 was 66,046 bp and that of Phage S2 was 94,434 bp. The phylogenetic tree indicated that the two phages were distantly related to each other and were classified in the genera *Pbunavirus* and *Pakpunavirus* respectively. Thirty-one proteins were identified for each phage by mass spectrometry and were used to substantiate the function of the predicted coding genes. The two phages inhibited the growth of *P. aeruginosa* strain PAO1 at low multiplicity of infection levels and had good performance both on preventing biofilm formation and eradicating preformed biofilms. They were also stable over a wide range of temperature and pH values, supporting their potential use in the treatment of *P. aeruginosa* infections.

## 1. Introduction

The recent emergence and the expanding distribution of multidrug-resistant (MDR), extensively drug-resistant, and pandrug-resistant bacterial strains have been a great challenge for public health due to the lack of effective antibiotic treatments. In particular, the ESKAPE organisms (*Enterococcus faecium*, *Staphylococcus aureus*, *Klebsiella pneumoniae*, *Acinetobacter baumannii*, *Pseudomonas aeruginosa*, and *Enterobacter species*) are the dominant causes of serious nosocomial infections [1,2,3]. As a ubiquitous Gram-negative opportunistic bacterium, *P. aeruginosa* can cause life-threatening infections in patients suffering from cystic fibrosis, severe burns, and other immunocompromising conditions, leading to considerable morbidity and mortality [4,5]. Unfortunately, MDR *P. aeruginosa* is difficult to eradicate by conventional antibiotics, owing to its sophisticated antibiotic resistance mechanisms, which includes intrinsic and acquired drug resistance, and its capacity to form biofilms [6,7,8]. Thus, there is an urgent demand for the development of alternative antibacterial strategies to combat these superbugs [9].

The interest in phages, which have been recognized as antimicrobial therapeutics for nearly a century, has recently resurged because of their high specificity and abundance [10,11,12,13]. Bacteriophages are natural predators of bacteria and are classified as virulent (lytic) phages or temperate (lysogenic) phages depending on their distinct life cycle. However, only virulent phages have been explored for phage therapy, mainly because temperate phages are associated to potential problems originating from their ability of transferring DNA between different host bacteria and the possible alteration of their host pathogenicity when shifting from quiescent state to lytic state [14,15]. Phage therapy has been proved a successful treatment option, not only in animal models [16] but also in human clinical trials [17]. Due to the high specificity of phages for a determined host, phage cocktails are often utilized in order to broaden the antibiotic spectrum of the therapy, which makes a request for exploiting and characterizing more therapeutic phages.

Recent advances in genome sequencing and biotechnology have greatly promoted the discovery and identification of numerous novel phages [18]. As for December 2018, the Genebank database has deposited 323 *Pseudomonas* phage genome sequences, among which more than two-thirds were *P. aeruginosa* phages. In order to be considered eligible for their use in phage therapy, *P. aeruginosa*-specific phages must be fully characterized to ensure that they are safe, i.e., their genomes must be studied in detail to prove the lack of genes encoding for toxins, virulence factors or other undesirable genes, through complete genome sequencing [19,20]. In this study, we characterized two *P. aeruginosa* phages, vB_PaeM_SCUT-S1 (Phage S1) and vB_PaeM_SCUT-S2 (Phage S2), at morphological, genomic, and proteomic levels. The two phages were new members of the genera *Pbunavirus* and *Pakpunavirus*, respectively, and their therapeutic potentials were investigated for the growth inhibitory effects on the planktonic cells and biofilm cells.

## 2. Materials and Methods

### 2.1. Bacterial Strains and Culture Conditions

The bacterial strains used in this study are listed in Table S1. *P. aeruginosa* ATCC 9027, ATCC 15442, ATCC 27853, and *Stenotrophomonas maltophilia* ATCC 51331 were purchased from Guangdong Microbial Culture Center, China (GDMCC). The strains PALWL1.001, PALWL1.002, and PALWL1.003 were collected by our laboratory. Strain PAO1 was a gift from Prof. Hu (Third Military Medical University, Chongqing, China). All bacterial strains and phages were routinely cultured at 37 °C in lysogeny broth (LB) containing 2 mM CaCl_2_. Phage plating was performed using the overlay agar method, with LB containing 0.6% and 1.5% agar used for the top and the base agar, respectively.

### 2.2. Bacteriophage Isolation, Propagation, and Purification

Phages were isolated by following the enrichment method using strain PAO1 as the host [21,22]. Briefly, aqueous samples were collected from a small pond in Guangzhou, China (N 23°03′7.12″, E 113°04′5.35″). After centrifugation for 10 min at 9000× *g*, the supernatant was filtered through a 0.45 μm filter and incubated with the host strains for 24 h at 37 °C. The bacterial cell debris was removed by centrifugation and filtration, and the supernatant was plated using the overlay agar method to check the host lysis. Twelve candidate plaques obtained were scraped with pipette tips and resuspended in SM buffer (100 mM NaCl, 8 mM MgSO_4_, and 50 mM Tris-HCl, pH 7.5), were then diluted 1000-fold in the same buffer, and purified by repeated co-culturing with the host strains using the overlay agar method (three rounds). The uniqueness of the phages was confirmed by analysis of the genome restriction maps obtained using three endonucleases (*Hind*III, *EcoR*I or *Xba*I) individually. Only two different phages were identified.

The phage propagation was performed according to the classic procedure [23]. An aliquot of 500 mL of PAO1 culture (oa. OD 0.4–0.6 at 600 nm) was infected by the pure phage stocks and grown overnight. Subsequently, 10 mL of chloroform were added, and the cells were incubated with shaking for 10 min at 37 °C to obtain the lysates. The genomic DNA of the host was removed by the treatment of 1 μg/mL DNase Ι and RNase for 30 min at room temperature. The lysate was then supplemented with a final concentration of 1 M NaCl and incubated for 1 h at 4 °C. After incubation and centrifugation, the supernatant was supplemented with 10% (*w*/*v*) PEG 8000 and stored at 4 °C overnight to precipitate the phage particles. After centrifugation (11,000× *g*, 10 min), the pellets were suspended in SM buffer and an equal volume of chloroform was added to extract the PEG and the bacterial debris. After centrifugation at 3000× *g* for 15 min at 4 °C, the suspension was adjusted by adding CsCl reagent to a density of 1.15 g/mL and carefully loaded on a CsCl density gradient consisting of the step gradient 1.35 g/mL, 1.5 g/mL, and 1.7 g/mL. After ultracentrifugation at 87,000× *g* for 2 h at 4 °C, the band of phage particles was collected and dialyzed twice for 1 h at 4 °C against a 500-fold volume of SM buffer to remove the remaining CsCl.

### 2.3. Host Range Analysis

Host range analysis was performed using the spot testing method [24] on three biological replicates. Briefly, 10 μL of phage suspensions of six concentrations ranging from 10^4^ to 10^9^ pfu/mL were spotted onto bacterial lawns and incubated at 37 °C for 24 h. After incubation, the spot morphology was observed and classified as “C+++”, a large lysis zone at 10^2^ pfu; “C++”, individual plaques at 10^2^–10^3^ pfu; “C+”, individual plaques at 10^4^–10^5^ pfu; “T”, a turbid lysis zone at 10^6^–10^7^ pfu; “-”, no lysis.

### 2.4. Electron Microscopy

For electron microscopy, the CsCl-purified phages were spotted onto 400 mesh carbon-coated grids and negatively stained with 2% phosphotungstic acid (pH 6.5) [25]. The grids were observed by a Hitachi transmission electron microscope at 80 kV. The dimensions of three viral particles of each phage was measured, and the values were averaged.

### 2.5. Temperature and pH Stability

The thermal stability testing was performed by incubating the phages (10^6^ pfu/mL) at different temperature for 1 h [26], the phage titers were then determined by using the double-layer overlay method. The relative titer was calculated as the ratio of phage titers at a different temperature to those stored at 4 °C. For the pH stability analysis, the phages (10^6^ pfu/mL) were diluted 100-fold with SM buffer of different pH values and incubated for 1 h at room temperature. The relative titer was calculated as the ratio of phage titers treated with different pH levels to those by the original SM buffer (pH7.5). Three independent experiments were performed.

### 2.6. Genome DNA Extraction and Sequencing

The genomic DNA was extracted as follows [23,27]. The purified phage was first treated with 2.5 U/mL DNase Ι and incubated at 37 °C for 1 h to remove the host DNA. Next, 0.1 volumes of 2 M Tris-HCl (pH 8.5)/0.2 M EDTA, 0.01 volumes of 0.5 M EDTA (pH 8.0), and 1 volume of formamide were added, and the solution was incubated for 30 min at room temperature. The raw genomic DNA was precipitated adding 2 volumes of 100% ethanol followed by centrifugation at 9000× *g* for 20 min at 4 °C. The DNA pellet was washed twice with 70% ethanol and suspended in 567 μL of TE buffer (10 mM Tris-HCl, 1 mM EDTA) and added with 30 μL of 10% (*w*/*v*) SDS and 3 μL of 20 mg/mL proteinase K. The mixture was incubated for 1 h at 50 °C. Subsequently, 100 μL of 5 M NaCl and 80 μL of CTAB (cetyltrimethylammonium bromide)/NaCl solution were added and incubated for 10 min at 65 °C. Next, the mixture was sequentially treated with one volume of chloroform, one volume of phenol/chloroform/isoamyl alcohol (25:24:1), and one volume of chloroform, to gradually purify the genomic DNA. The aqueous phase was collected, and 0.7 volumes of isopropanol were added to precipitate the DNA. After centrifugation at 13,000× *g* for 15 min at 4 °C, the purified DNA pellet was washed twice with 500 μL of ice-cold 70% ethanol and left to dry. The air-dried pellets were suspended in 20 μL TE. The DNA concentration was measured by a Nanodrop2000 (Thermo Fisher Scientific, Waltham, MA, USA). The purified DNA was used for the whole genome sequencing by Personal Biotechnology Corp. (Shanghai, China). A DNA library with an insert size of 400 bp was prepared for each sample. The two phage samples were sequenced by an Illumina MiSeq platform using the PE 250bp strategy.

### 2.7. Genome Assembly, Annotation, and Comparison

After filtering the raw reads to remove the adaptor contamination (i.e., low-quality reads with ‘N’ bases), the remaining high-quality reads were assembled by ABYSS 2.0.2 [28] and MIRA 4.0 [29]. The contigs were manually assembled based on overlaps of more than 40 bp to obtain the final scaffold. Next, the coverage was visualized using Geneious 10.2.3 to identify the genome termini, and the results were confirmed using PhageTerm [30], which is available on the public Galaxy server (http://galaxy.pasteur.fr/). After the whole genome sequences were obtained, the annotation was carried out using RAST [31,32,33], Glimmer [34], and GeneMarkS [35], then was subsequently manually confirmed. In addition, tRNAs were predicted by tRNA-Scan [36] and ARAGORN (http://mbio-serv2.mbioekol.lu.se/ARAGORN/). The complete genome sequences of Phages S1 and S2 were searched for similarity against the reported genomes by BLASTN https://www.ncbi.nlm.nih.gov/BLAST/). Next, the genomes of the five most similar phages were re-annotated by the same procedure as that of the two phages, and the transcribed ORF sequences were compared by BLASTP. Comparative analysis of the whole genomes was performed by MAFFT [37]. The conserved sequences were extracted by Gblocks [38] and then used to construct a maximum likelihood tree by RAxML [39]. The phylogenetic trees based on the amino acid sequences of the terminase large subunit were constructed using Molecular Evolutionary Genetic Analysis (MEGA) 7.0 [40]. All the phylogenetic trees were visualized by MEGA 7.0.

### 2.8. Proteomics Analysis

The structural proteins of the phages were analyzed after removing the DNA, as previously described [41]. Briefly, the purified phage particles were mixed with an equal volume of 10 M LiCl and incubated for 10 min at 46 °C. Next, the mixture was diluted 10-fold with SM buffer, followed by 10 mM MgCl_2_ and 50 U DNase Ι per 10^12^ pfu. After 2 h incubation at 37 °C, the “ghost” particles were precipitated by ultracentrifugation at 100,000× *g* at 4 °C for 30 min. Subsequently, the phage pellet was suspended in protein loading buffer and denatured at 95 °C for 10 min. The phage proteins were separated on a 12% SDS-PAGE gel and stained with Coomassie Brilliant Blue. For the protein identification, the whole gel lane was cut, and the peptide mixture obtained by in-gel trypsin digestion was analyzed by nanoLC-MS/MS (Eksigent nanoLC-Ultra 2D and TripleTOF 5600, AB SCIEX). The MS/MS data were analyzed by MASCOT (http://www.matrixscience.com/) using the predicted proteins of Phage S1 or S2 as the databases. The phage proteins were identified using a minimum threshold of two peptides per protein.

### 2.9. One-Step Growth Curve and Lysis Kinetics

A 0.1 mL aliquot of phage suspension (10^7^ pfu/mL) was added to 9.9 mL of mid-log-phase bacterial culture of host strain PAO1 (ca. OD_600_ ~ 0.5) and incubated for 5 min [42]. Phage titers were detected by collecting samples at 5- or 10-min intervals and plating them immediately by the overlay method. The average burst size was quantified as the difference between the final and the initial phage titer divided by the initial phage titer.

To measure the lytic kinetics, phages with varying multiplicity of infection (MOI) in the range 0.01–100 were incubated with mid-log-phase bacterial cultures of host strain PAO1 (ca. OD_600_ ~ 0.5) in 96-well microtiter plates at 37 °C, 180 rpm. The kinetic data were obtained by monitoring the change of absorbance at 600 nm for 20 h with intervals of 30 min using a microplate reader (Tecan infinite M200PRO, Zürich, Switzerland).

### 2.10. Biofilm Inhibition Assays

Biofilms were grown in 96-well microplates as previously described [43], with slight modifications. Briefly, an overnight-grown PAO1 culture was diluted 1:100 with fresh tryptic soy broth medium (TSB, 17 g of pancreatic digest of casein, 3 g of papaic digest of soya bean, 5 g of NaCl, 2.5 g of K_2_HPO_4_, and 2.5 g of glucose monohydrate for a 1 L solution, with a final pH adjusted to 7.3), and 150 μL aliquots of this diluted culture were transferred into the wells of round-bottomed microplates (polystyrene) and incubated under a static condition at 37 °C. To analyze the capacity of preventing biofilm formation, 50 μL of phage suspensions (approximately 10^8^ pfu/well) in TSB medium or 50 μL of TSB medium without phages as the controls were added to each well, and the plates were incubated for different time periods (4, 8, and 24 h). To analyze the capacity of removing preformed biofilms, the plates were incubated for 24 h to form the biofilms and rinsed three times with 0.9% NaCl to remove the planktonic cells. Next, 200 μL of phage suspensions (approximately 10^8^ pfu/well) in TSB medium, or 200 μL of TSB medium without phages as the controls, were added to each well and were incubated at 37 °C for different time periods (4, 8, and 24 h). The plates were all washed three times with 0.9% NaCl for the next assays.

CV (crystal violet) staining or XTT assay was performed to evaluate the biofilm conditions. For the CV staining, the biofilms were stained with 220 μL of 0.1% crystal violet solution for 10 min, then washed three times with 0.9% NaCl to remove the excess of CV, and left to dry in the air. Next, 220 μL of 30% acetic acid were added to dissolve the bound CV. The eluted stain was transferred into another microplate, and the absorbance was measured at 590 nm [44]. For the XTT assay, 220 μL of a solution containing 0.5 mg/mL XTT and 50 μM menadione were added to the wells containing the rinsed biofilms. After incubation in the dark for 2 h at 37 °C, the solution was then transferred into another microplate to measure the absorbance at 460 nm [45].

### 2.11. Accession Number

The whole genome sequences of phage vB_PaeM_SCUT-S1 and vB_PaeM_SCUT-S2 were deposited in GenBank under the accession number MK340760 and MK340761, respectively.

## 3. Results

### 3.1. Isolation and Characterization of the Phages

**Figure 1:**
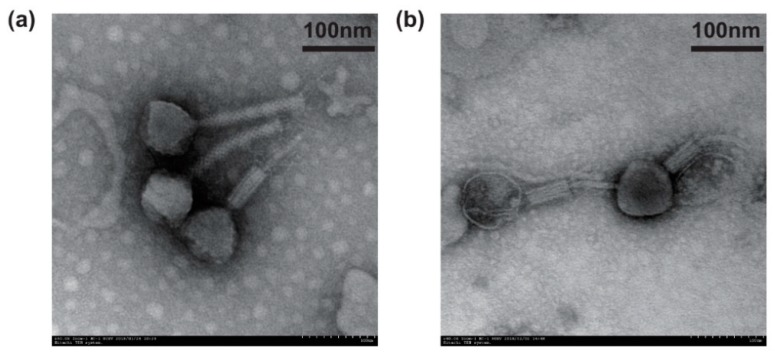
Transmission electron microscopy images of phages (**a**) vB_PaeM_SCUT-S1 and (**b**) vB_ PaeM_SCUT-S2 negatively stained by 2% phosphotungstic acid.

Using *P. aeruginosa* PAO1 as the host strain, we isolated two phages from aquatic environment samples. Both the phages formed clear plaques in the double agar layer lawn (Figure S1), which indicated they were virulent phages. The spotting test showed that the two phages could infect most *P. aeruginosa* strains with large and clear lysis zones (Table S1). From the electron microscopic imaging, it was observed that both phages had icosahedral heads and contractile tails (Figure 1a,b), which suggested that they both belonged to the order *Caudovirales* and the *Myoviridae* family. Thus, we designated them as vB_PaeM_SCUT-S1 and vB_PaeM_SCUT-S2 (hereinafter referred to as S1 and S2, respectively) according to the ICTV nomenclature for virus. Phage S1 had a capsid size of 77 ± 2 nm in diameter and a tail length of 154 ± 2 nm, while Phage S2 had a capsid size of 85 ± 3 nm in diameter and a tail length of 136 ± 3 nm (Figure 1a,b).

### 3.2. Basic Characteristics of the Genomes

**Figure 2:**
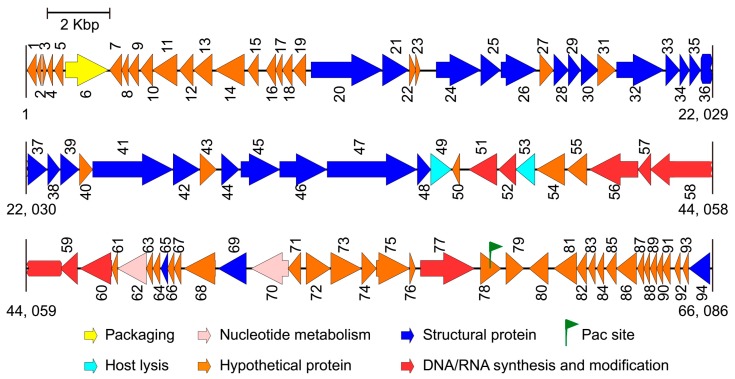
Genome map of phage vB_PaeM_SCUT-S1. The arrows indicate the predicted open reading frames, and the different colors indicate the diverse functions of the coded proteins.

**Figure 3:**
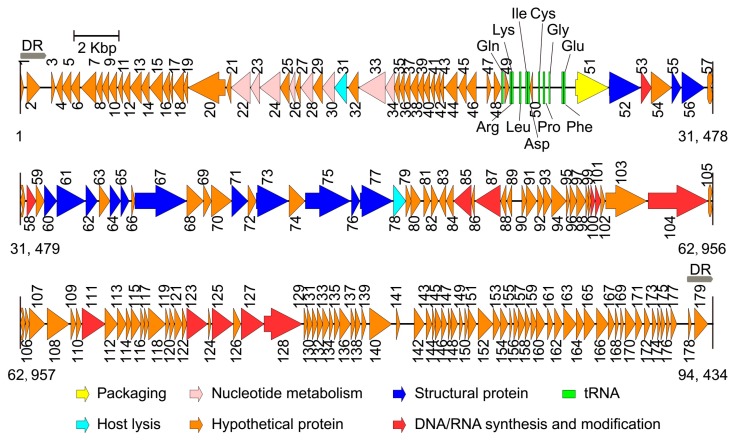
Genome map of phage vB_PaeM_SCUT-S2. The arrows indicate the predicted open reading frames, and the different colors indicate the diverse functions of the coded proteins. DR: direct repeat.

After performing the genome sequencing and data processing, high-quality, 578 and 424 Mbp sequences were obtained for Phages S1 and S2, and the average genome coverage was about ~8700 and ~4500, respectively. After performing the assembly and manually refinement, we obtained the complete genomes of the two phages. For Phage S1, the genome had a size of 66,089 bp and a G + C content of 55.43%. The genome termini were identified as circularly permuted by assessing the sequence coverage using PhageTerm. The predicted Pac site was located in an AT-rich region, which could be a replication origin (Figure 2). The genome was tightly organized, and the coding regions were about 93% of the whole genome with 94 predicted ORFs (Figure 2 and Table S2). None of the tRNA prediction tools used was able to identify any tRNA genes, indicating that Phage S1 is likely to exploit the host tRNA machinery for its protein synthesis. For Phage S2, the complete genome size was 94,434 bp with direct repeats (DRs) of 1189 bp at the ends. There were 197 ORFs identified for Phage S2 (Figure 3 and Table S3). Phage S2 had a much lower G + C content (49.34%) than that of the host strain PAO1 (66.3%), which was indicative of differences in the codon usage between the phage and its host. Accordingly, a tRNA cluster of 11 tRNA genes was predicted (Figure 3).

The proteins identified for the two phages could be categorized into five functional classes: nucleotide metabolism and DNA replication (including DNA repair and modification), virion structure (including capsid and tail morphogenesis), DNA packaging, host lysis, and hypothetical proteins (Figure 2 and Figure 3; Tables S2 and S3). The structural protein genes were well clustered, and the nucleotide metabolism and replication-related genes neighbored each other. In addition, three and five clusters of unknown function were annotated for Phages S1 and S2 (i.e., ORF7-19, ORF71-76, and ORF78-93 for Phage S1 and ORF1-21, ORF34-49, ORF79-99, ORF105-121, and ORF129-179 for Phage S2), respectively. No integrase, excisionase, and repressor genes, which are considered indicative of potential for a lysogenic cycle, were found in the two genomes, supporting the conclusion that both S1 and S2 are lytic phages.

### 3.3. Comparative Genome Analysis

**Figure 4:**
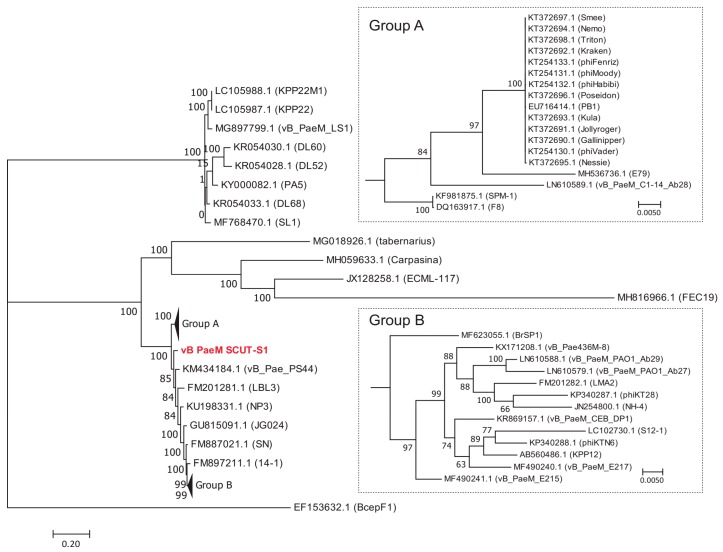
Maximum likelihood tree showing the phylogenetic relationships among phages of genus *Pbunavirus*. The whole genome sequences were aligned by MAFFT [37], and the tree was visualized by MEGA 7 [40]. The value at the nodes indicated the bootstrap support scores as calculated using 1000 replicates. Phage vB_PaeM_SCUT-S1 was colored red.

**Figure 5:**
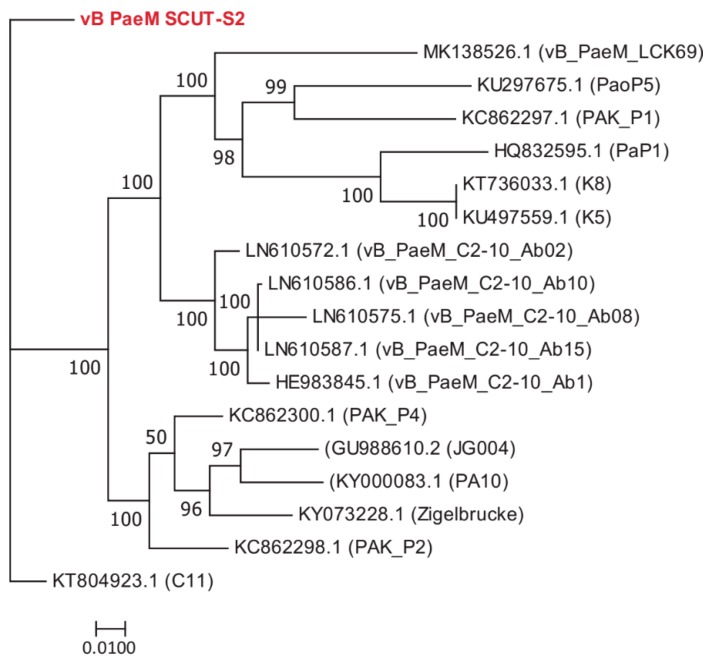
Maximum likelihood tree showing the phylogenetic relationships among phages of genus *Pakpunavirus*. The whole genome sequences were aligned by MAFFT [37] and the tree was visualized by MEGA 7 [40]. The value at the nodes indicated the bootstrap support scores as calculated using 1000 replicates. Phage vB_PaeM_SCUT-S2 was colored red.

The BLASTN results indicated that Phages S1 and S2 were homologous to the viruses of genera *Pbunavirus* and *Pakpunavirus*, with the nucleotide similarity to congeneric phages varying from 68.56–94.00% and 94.97–97.99%, respectively. Based on these results, we compared the amino acid identity of the two phages to their five most similar phages by BLASTP analysis. Phage S1 harbored two or more unique ORFs, and Phage S2 had six or more unique ORFs (Table S4). There were 50 and 22 complete genome sequences for genus *Pbunavirus* (taxid:1198980) and *Pakpunavirus* (taxid:1921407) in the Genbank database (as to December 2018), respectively. Thus, the intra-genus phylogenetic location was explored at a genomic scale. For Phage S1, no phages were found at the same evolutionary level. Phage S1 had the highest homology with phage vB_Pae_PS44 (KM434184.1) isolated in Poland and phage LBL3 (FM201281.1) isolated in Belgium, and was more distantly related to *Burkholderia ambifaria* phage BcepF1 (EF153632.1), isolated in the USA, and *Escherichia* phage FEC19 (MH816966.1), isolated in China (Figure 4). For the phages of *Pakpunavirus*, two main distant clades were clustered, the smallest of which consisted of five phages. To better illustrate the phylogeny of Phage S2, the members of the largest clade were selected and used to construct the maximum likelihood tree. Phage S2 was located at a distinct branch, with a close relationship to phage C11 (KT804923.1) isolated in China and distant to the other phages, which is indicative of the low homology of Phage S2 to the other *Pakpunavirus* (Figure 5). Since the comparative genome analysis indicated that Phages S1 and S2 are new members of the genera *Pbunavirus* and *Pakpunavirus*, respectively, the phylogenetic relationships among *Pseudomonas* phages of the *Myoviridae* family were explored based on the terminase large subunit (Figure S3). Most phages clustered in five groups, especially the *Pbunavirus* group (Green) and *Pakpunavirus* group (Brown) (Figure S3). *Pbunaviruses* showed a distant relationship to the other groups, separated by some phages in the scattered clades (Figure S3).

### 3.4. Structural Proteins of the Two Phages

To better understand the functions of the annotated genes, we performed a proteomic analysis of the two phages. After the genomic DNAs of the purified phage particles were released by LiCl, the phages proteins were separated by SDS-PAGE on a 12% gel. The protein profiles of the two phages were different due to their distinct genomes (Figure S2). For Phage S1, at least 16 protein bands were detected with a molecular mass ranging from 12 to 100 kDa (Figure S2a). About 14 protein bands within the range from 14 to 90 kDa were detected for Phage S2 (Figure S2b). In order to identify low abundance proteins that might not be detected by Coomassie blue staining, the whole lane of the gels was cut and analyzed by nanoLC-MS/MS instead of each stained band. Using the predicted ORFs as the searching database, 31 proteins with at least two detected peptides were identified for the two phages (Table 1 and Table 2). For most of the identified proteins (i.e., 27/31 for Phage S1 and 20/31 for Phage S2), the sequence coverage was above 20% (Table 1 and Table 2), which indicated the high confidence of the peptides. We speculated that ORF22, ORF27, ORF31, and ORF54 of Phage S1, and ORF57, ORF59, ORF63, ORF68, ORF69, ORF70, ORF72, ORF74, and ORF80 of Phage S2 could be virion structural proteins, based on the vicinity of their genes to genes coding for structural proteins. For ORF11, ORF72, and ORF84 of Phage S1, and ORF14, ORF80, and ORF140 of Phage S2, no function could be assigned.

The first step of bacteria elimination by phages is their successful invasion into the host cells [46,47]. During this process, the tail fiber proteins are believed to play an important role in host recognition, and a single amino acid mutation can change the host specificity and give the phage the capacity of infecting different species of bacteria [48,49]. Thus, we compared the predicted tail fiber proteins of the five closest phages based on the terminase large subunit (Figure 4 and Figure 5). For the tail fiber proteins of Phage S1 and related phages, ORF33 presented only few amino acid differences and ORF47 showed some variations at the C-terminus (Figure S4a). In the case of Phage S2 and related phages, ORF75 presented low identity at the C-terminus and ORF77 showed some amino acid mutations (Figure S4b). Interestingly, several proteins involved in nucleotide metabolism and DNA modification were detected in the phages particles, such as putative DNA helicase (ORF56 of S1), putative RNA polymerase (ORF58 of S2), putative ATP-dependent exonuclease V (ORF70 of S1), putative ribonucleoside-diphosphate reductase alpha and beta subunits (ORF127 and ORF128 of S2), putative 3’-phosphatase (ORF123 of S2), putative nictotinate phosphoribosyltransferase (ORF20 of S2), and putative methyltransferase (ORF53 of S2) (Table 1 and Table 2), which indicate a potential role of these proteins in the phage infection mechanism.

### 3.5. Stability Analysis

**Figure 6:**
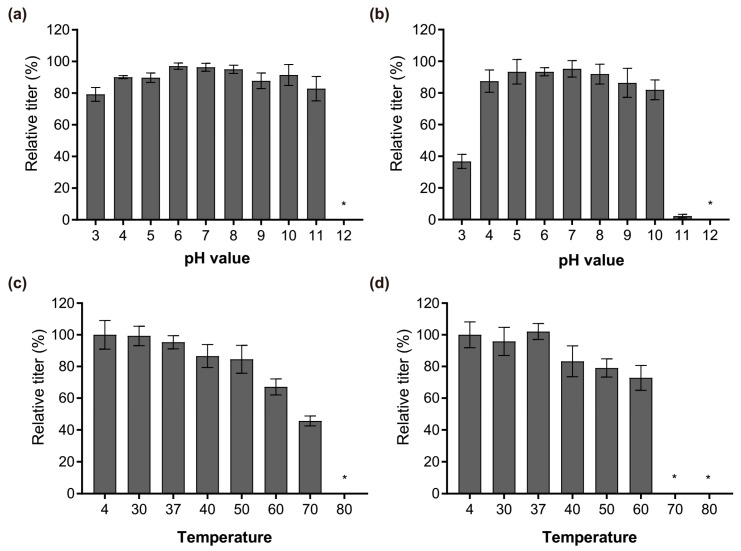
Relative amount of infectious phage particles after different treatments. (**a**) vB_PaeM_SCUT-S1 and (**b**) vB_PaeM_SCUT-S2 incubated for 1 h at different pH levels; (**c**) vB_PaeM_SCUT-S1 and (**d**) vB_PaeM_SCUT-S2 incubated for 1 h at different temperatures. * No plaques were detected at pH 12 for either Phages S1 and S2 (**a**,**b**), at 80 °C for Phage S1 (**c**) and at 70 °C or 80 °C for Phage S2 (**d**), indicating that the phages were completely inactive in these conditions. Three independent experiments were performed.

After the characterization at genomic and proteomic level, the physiological properties of Phages S1 and S2, in terms of pH and temperature stability, were investigated. Phage S1 was more robust to pH changes than Phage S2 was. Their infectivity both remained almost intact when exposed for 1 h to a pH ranging from 4 to 10 (Figure 6a,b). However, the performance changed when exposed to the cruel pH conditions. The infectivity of Phage S1 slightly decreased at pH 3 and pH 11 (Figure 6a), while that of Phage S2 decreased significantly at pH 3 and nearly lost at pH 11 (Figure 6b). The two phages were both inactivated when exposed to pH 12 (Figure 6a,b). Regarding the temperature stability, the two phages also showed different properties. The infectivity of Phage S1 decreased gradually upon increasing the temperature when exposed for 1 h to temperatures ranging from 30 to 60 °C, dropped significantly at 70 °C, and was completely lost at 80 °C (Figure 6c). Conversely, the infectivity of Phage S2 showed a slight decrease upon increasing the temperature up to 60 °C but was completely lost at 70 °C or above (Figure 6d). The different response of the two phages to the temperature could result from their distinct capsid proteins or structures.

### 3.6. Growth Characteristics and Lysis Kinetics

**Figure 7:**
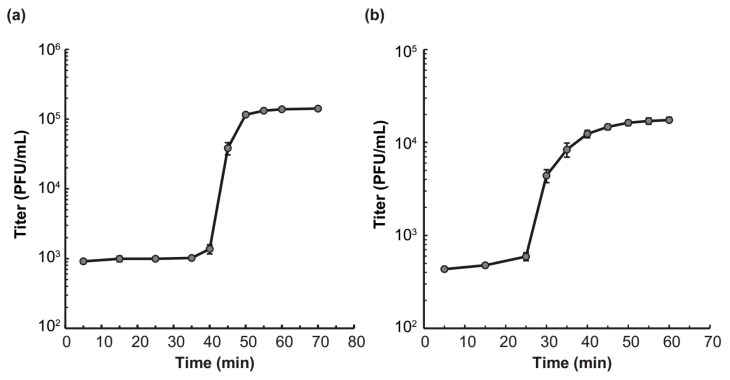
One step growth curve of (**a**) vB_PaeM_SCUT-S1 and (**b**) vB_PaeM_SCUT-S1. Data are presented as the mean (± standard deviations) titers measured at the indicated infection time obtained from three independent experiments.

The one-step growth curve analysis revealed that Phages S1 and S2 had distinctive life cycles (Figure 7a,b). Phage S1 had a latent period of 40 min and a rise period of 10 min, and generated about 134 virion progenies per infected cell (Figure 7a). Phage S2 had a shorter latent period and a prolonged rise period, both being about 25 min, and the average burst size was much lower (i.e., 40 progenies per infected cell) than that of Phage S1 (Figure 7b). These differences in growth kinetics can result in different host lysis efficiency.

**Figure 8:**
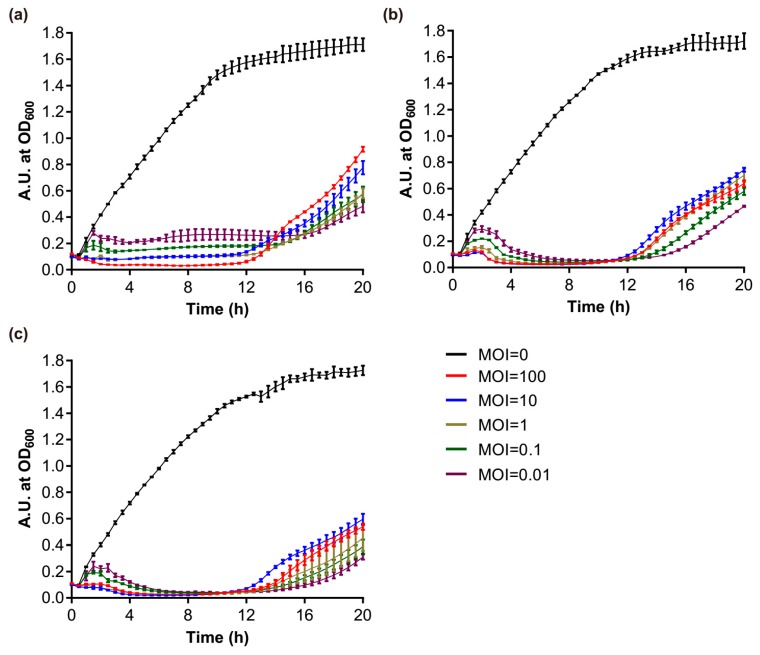
Growth curves of PAO1 strains infected with different phages. (**a**) vB_PaeM_SCUT-S1 of different MOIs; (**b**) vB_PaeM_SCUT-S2 of different MOIs; (**c**) vB_PaeM_SCUT-S1 and vB_PaeM_SCUT-S2 were combined in a 1:1 ratio to the indicated final MOIs.

The effect of S1 and S2 against *P. aeruginosa* PAO1 planktonic cultures were determined by using the phages alone or in combination. Phages S1 and S2 efficiently inhibited the growth of the host bacteria at the exponential phase. The number of phages had a great influence on bacterial growth in the early stage, and at the highest titer (i.e., MOI = 100) the phages could kill the host cells directly without needing a latent period to produce further virions (Figure 8a,b). As the growth time elapsed, this advantage of higher phage titers gradually disappeared. The bacterial growth was totally inhibited by Phage S2 after 3.5 h for the different treatments, including the lowest MOI = 0.01 (Figure 8b). The inhibitory effect of Phage S1 was slightly lower than Phage S2 (Figure 8a). The host lysis became inefficient after 12 h of culture, and the higher the MOI used at the beginning, the higher the bacteria grown at 20 h (Figure 8a,b), which suggested the emergence of phage-insensitive or phage-resistant strains. This phenomenon was not alleviated by using the two phages in combination (Figure 8c). The lysis curve observed using a 1:1 mixture of Phages S1 and S2 was very similar to that obtained using Phage S2 alone, indicating that the lysis induced by Phage S1 was nearly covered by that of Phage S2.

### 3.7. Biofilm Eradication

**Figure 9:**
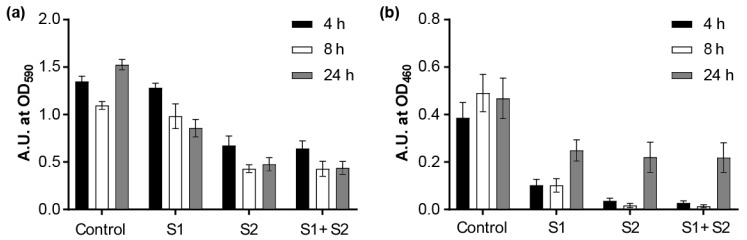
Effect of phage treatment on 24-h-grown biofilms. (**a**) Biomass evaluation by CV staining; (**b**) metabolic activity evaluation by XTT assay. Control: without any phages; S1: treated with 10^8^ pfu of phage vB_PaeM_SCUT-S1; S2: treated with 10^8^ pfu phage vB_PaeM_SCUT-S2; S1 + S2: treated with a mixture of vB_PaeM_SCUT-S1 and vB_PaeM_SCUT-S2 (0.5 × 10^8^ pfu of each phage). The different treatment duration is indicated by different bar colors.

We first tested whether the phages could prevent the biofilm formation based on the biofilm biomass, which was tested by CV staining. The results showed that the phages alone or in combination could effectively inhibit the biofilm growth upon incubation for 4, 8, and 24 h (Figure S5). Next, the biofilm-degradation capacity of Phages S1 and S2 was evaluated by investigating two complementary biofilm properties: the biofilm biomass (tested by CV staining) and the cell metabolic activity (tested by XTT assay) (Figure 9). When the 24-h-grown PAO1 biofilms were treated with the phages for different time periods (4, 8, and 24 h), the biofilm was effectively eradicated by either phage used alone as well as by the combination of the two phages (Figure 9). The biomass of the biofilm was reduced more effectively by prolonged treatment. The relative reduction was 5% at 4 h, 10% at 8 h, and 44% at 24 h for Phage S1 and 50% at 4 h, 61% at 8 h, and 69% at 24 h for Phage S2 (Figure 9a). The combination of Phages S1 and S2 showed nearly the same reduction observed when using Phage S2 alone (Figure 9a). Regarding the cell metabolic activity, the 8 h treatment provided the most effective inhibition (i.e., 79% for Phage S1 and 97% for both Phage S2 and the combination of Phages S1 and S2, Figure 9b), while upon 24 h treatment, a significant recovery of metabolic activity was observed (Figure 9b). The observed biofilm metabolic activity was more consistent with the measured lysis kinetics than the biofilm biomass (see paragraph 3.6). Overall, Phage S2 showed a better performance on growth inhibition and biofilm reduction than Phage S1, and the combination of Phages S1 and S2 did not show an obvious synergistic effect in either planktonic cells or biofilm eradication.

## 4. Discussion

The identification and characterization of novel phages can enhance our understanding of phage biology. Phages S1 and S2, which were assigned to the *Myoviridae* family, have genome sizes of 66.1 kb and 94.4 kb encoding 94 and 179 putative proteins, respectively. Both phages showed genetic mosaicism, a typical feature of tailed phages [50], with several functional modules clustered throughout their genomes (Figure 2 and Figure 3). In addition to a number of clustered genes coding for proteins with predictable function, several ORFs could only be annotated as coding for proteins with unknown function (Tables S2 and S3), a common problem in phage studies [51]. Based on our proteomic analysis, 23 and 21 ORFs were identified as structural protein coding genes for Phages S1 and S2, respectively (Table 1 and Table 2), a result that contributes to the enrichment of phage proteome collection. Although Phages S1 and S2 were isolated from the same location, they belonged to two different genera, *Pbunavirus* and *Pakpunavirus*, respectively. Based on the phylogenetic analysis, they were found to be distantly related to each other (Figure S3), suggesting that the two phages evolved independently and might have different roles in regulating bacterial communities. This is consistent with a recent study in which several phages where isolated from a single environmental source [52]. Interestingly, Phage S2 presented a dominant advantage over Phage S1 in infecting PAO1 strain and in reducing its biofilm, which inspires for a better exploration of the relationships between the other phages in the genera *Pbunavirus* and *Pakpunavirus*.

The two phages could be suitable candidates to be further developed for phage therapy. In fact, Phages S1 and S2 are virulent phages with a conventional genome size and no genes coding for toxins, virulence factors, or lysogeny-related proteins were discovered by genome annotation and proteomic analysis (Figure 2 and Figure 3, Table 1 and Table 2 and Tables S2 and S3), in contrast to lysogenic phages, which are not recommended for therapy [53]. The infectivity of the phages remained stable in a temperature range of 4–37 °C and a pH range of 4–10 (Figure 6), indicating that they could be stored at room temperature and the activity could be maintained in a human physiological condition. Furthermore, they inhibited the growth of planktonic cells effectively alone or together at a low MOI for a 12 h treatment (Figure 8) and had good performances on preventing the biofilm formation (Figure S5) and eradicating biofilms (Figure 9). However, particular attention should be paid to the potential development of phage resistance in clinical settings since the bacterial growth could be partially recovered with a 24 h treatment in vitro (Figure 8 and Figure 9). Although the development of bacterial resistance considered inevitable in phage therapy, it is believed to occur less infrequently in vivo than in vitro [54]. In addition, phage adaptation or training performed by in vitro procedures could be utilized to solve this problem [54,55]. For example, the short-term antagonistic evolution of the *P. aeruginosa* strain PAO1 and the *Pbunaviruses* phage KPP22 resulted in the formation of KPP22 mutants with recovered infectivity towards phage-resistant PAO1 clones [56].

Members of genera *Pbunavirus* and *Pakpunavirus* have been studied for their suitability as therapeutics, and several phages have already been proved to be effective in controlling *P. aeruginosa* MDR infections in animal models [57,58,59,60,61,62]. For example, *Pbunaviruses Φ*NH-4 could effectively clear the *Pseudomonas* infection of murine lungs in 6 h [59], and a cocktail of three *Pbunaviruses* DL52, DL60, DL68 and other three *Podoviridae* phages could prolong survival of *Galleria mellonella* infected by clinical strains of *P. aeruginosa* [60]. *Pakpunavirus* PAK_P1 was effective in treating acute lung infection in a mouse model [61] and six closely related *Pakpunavirus* phages gave high survival rates, between 75 and 100%, with a 13-day treatment in a mouse lung infection model [62]. Phages S1 and S2 harbored distinctive fiber proteins (Figure S4) and might give rise to unique host recognition ranges [48,49]. Consequently, Phages S1 and S2 can have a host range different from that of previously reported phages [21,63,64] (e.g., both phages could infect 6/7 host *P. aeruginosa* strains in our study (Table S1)), which makes them potential candidates for their inclusion in phage therapy cocktails. Further research will study their efficacy in controlling clinical *P. aeruginosa* MDR infections and confirm the safety of the two phages as therapeutic tools.

## Figures and Tables

**Table 1:** Genes encoding virion proteins in vB_PaeM_SCUT-S1 identified by mass spectrometry.

No.	Predicted Function	Gene No.	Mol. Mass (kDa)	No. of Peptides	Coverage
1	putative tail protein containing transglycosylase	ORF41	94.39	72	81%
2	putative tail fiber protein	ORF47	103.16	44	63%
3	putative minor head protein	ORF20	84.53	44	59%
4	putative structural protein	ORF46	54.81	23	60%
5	putative major structural protein	ORF26	41.59	20	49%
6	putative structural protein	ORF32	53.82	19	47%
7	putative minor head protein	ORF21	31.72	16	56%
8	putative structural protein	ORF24	52.10	14	38%
9	putative tail fiber protein	ORF33	15.91	12	70%
10	putative structural protein	ORF42	32.60	12	58%
11	putative structural protein	ORF31	21.48	12	77%
12	putative baseplate protein	ORF45	43.51	11	41%
13	putative endolysin	ORF49	24.35	11	48%
14	putative structural protein	ORF25	21.57	9	59%
15	putative structural protein	ORF38	19.94	9	60%
16	putative structural protein	ORF54	32.21	8	25%
17	putative structural protein	ORF37	17.80	7	67%
18	putative structural protein	ORF28	16.96	7	30%
19	putative structural protein	ORF22	7.46	7	77%
20	putative tail fiber protein	ORF39	21.70	6	44%
21	putative structural protein	ORF36	14.46	6	50%
22	putative structural protein	ORF35	12.74	5	29%
23	unknown function protein	ORF84	8.61	4	47%
24	putative holin	ORF53	21.13	4	25%
25	putative structural protein	ORF27	16.37	4	22%
26	putative DNA helicase	ORF56	59.43	4	7%
27	putative structural protein	ORF34	12.36	3	42%
28	putative ATP-dependent exonuclease V	ORF70	45.54	3	7%
29	putative baseplate protein	ORF44	24.17	3	13%
30	unknown function protein	ORF72	29.47	3	10%
31	unknown function protein	ORF11	15.52	2	21%

**Table 2:** Genes encoding virion proteins in vB_PaeM_SCUT-S2 identified by mass spectrometry.

No.	Predicted Function	Gene No.	Mol. Mass (kDa)	No. of Peptides	Coverage
1	putative tape measure protein	ORF67	85.86	55	66%
2	putative structural protein	ORF52	54.49	38	77%
3	putative structural protein	ORF61	46.35	31	51%
4	major capsid protein	ORF56	39.41	30	72%
5	putative tail fiber protein	ORF75	71.97	29	69%
6	putative tail fiber protein	ORF77	53.15	23	58%
7	putative baseplate component	ORF73	52.37	17	36%
8	putative structural protein	ORF74	26.64	17	80%
9	putative structural protein	ORF55	14.85	14	90%
10	putative baseplate protein	ORF71	26.84	11	53%
11	putative structural protein	ORF60	21.27	10	43%
12	putative structural protein	ORF70	34.17	10	35%
13	putative structural protein	ORF68	28.60	10	49%
14	putative structural protein	ORF57	18.24	9	40%
15	putative structural protein	ORF62	18.95	8	55%
16	putative structural protein	ORF63	18.29	8	40%
17	putative structural protein	ORF72	14.07	7	32%
18	putative structural protein	ORF59	14.46	7	73%
19	unknown function protein	ORF108	37.10	6	17%
20	putative RNA polymerase	ORF58	15.51	5	15%
21	putative structural protein	ORF80	16.06	4	32%
22	putative endolysin	ORF78	20.89	4	20%
23	putative ribonucleoside-diphosphate reductase alpha chain	ORF128	67.42	4	6%
24	putative ribonucleoside-diphosphate reductase beta subunit	ORF127	40.62	4	8%
25	putative 3’-phosphatase	ORF123	35.43	3	9%
26	putative nictotinate phosphoribosyltransferase	ORF20	63.05	3	5%
27	unknown function protein	ORF14	14.86	3	6%
28	putative structural protein	ORF64	17.75	2	12%
29	putative methyltransferase	ORF53	17.27	2	14%
30	putative structural protein	ORF69	13.98	2	20%
31	unknown function protein	ORF140	37.78	2	4%

## Supplementary Materials

The following are available online at https://www.mdpi.com/1999-4915/11/4/318/s1. Figure S1: Plaques formed by phage vB_PaeM_SCUT-S1 (a) and vB_PaeM_SCUT-S2 (b) infecting *P. aeruginosa* PAO1. Figure S2: Identification of the structural proteins by SDS–PAGE analysis. Figure S3: Neighbor-joining tree showing the phylogenetic relationships among *Pseudomonas* phages of *Myoviridae* family. Figure S4: The comparison of the tail fiber proteins of phage vB_PaeM_SCUT-S1 (a) and vB_PaeM_SCUT-S2 (b) to the five most closely related phages. Figure S5. The inhibitory effect of phage treatment on biofilm formation based on the biomass tested by CV staining. Table S1: Host range of phage vB_PaeM_SCUT-S1 and vB_PaeM_SCUT-S2. Table S2: The genome annotation of *Pseudomonas* phage vB_PaeM_SCUT-S1. Table S3: The genome annotation of *Pseudomonas* phage vB_PaeM_SCUT-S2. Table S4: Amino acid identity between phage vB_PaeM_SCUT-S1 (a) and phage vB_PaeM_SCUT-S2 (b) with five most similar members indicated by BLASTN analysis.
