# Supplementary material for: Characterization of Two Pseudomonas aeruginosa Viruses vB_PaeM_SCUT-S1 and vB_PaeM_SCUT-S2
Source: Viruses. 2019 Apr 1;11(4):318. doi: 10.3390/v11040318 (PMC6521218; doi:10.3390/v11040318)
Supplement: Supplementary file 1 [file viruses-11-00318-s001.pdf]

Supporting information

Characterization of two *Pseudomonas aeruginosa* viruses vB_PaeM_SCUT-S1 and vB_PaeM_SCUT-S2

Yangyijun Guo [1], Ping Chen [1], Zhanglin Lin [1,*] and Tingting Wang [1,*]

[1] School of Biology and Biological Engineering, South China University of Technology, 382 East Outer Loop Road, Higher Education Mega Centre, Guangzhou, Guangdong 510006, China;

* Correspondence: zhanglinlin@scut.edu.cn (Z.L); wangtt@scut.edu.cn (T.W.); Tel.: +86-20-3839-0639, (T.W.)

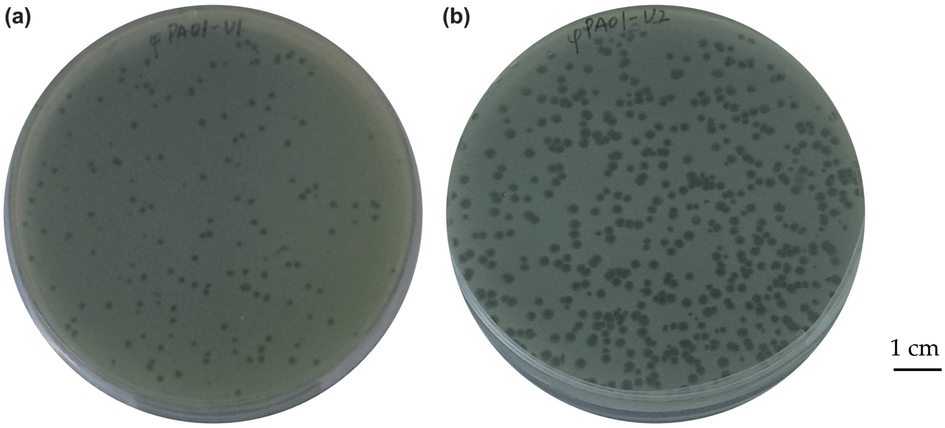

Figure S1. Plaques formed by phage vB_PaeM_SCUT-S1 (a) and vB_PaeM_SCUT-S2 (b) infecting *P. aeruginosa* PAO1.

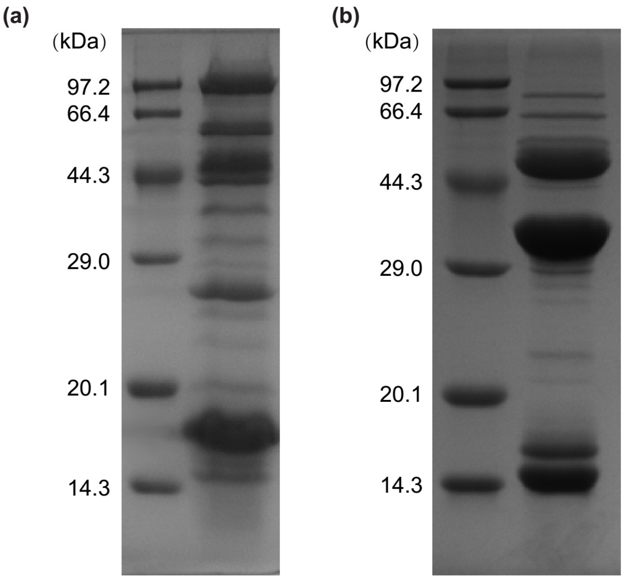

Figure S2. Identification of the structural proteins. (a) SDS–PAGE analysis of phage vB_PaeM_SCUT-S1; (b) SDS–PAGE analysis of phage vB_PaeM_SCUT-S2.

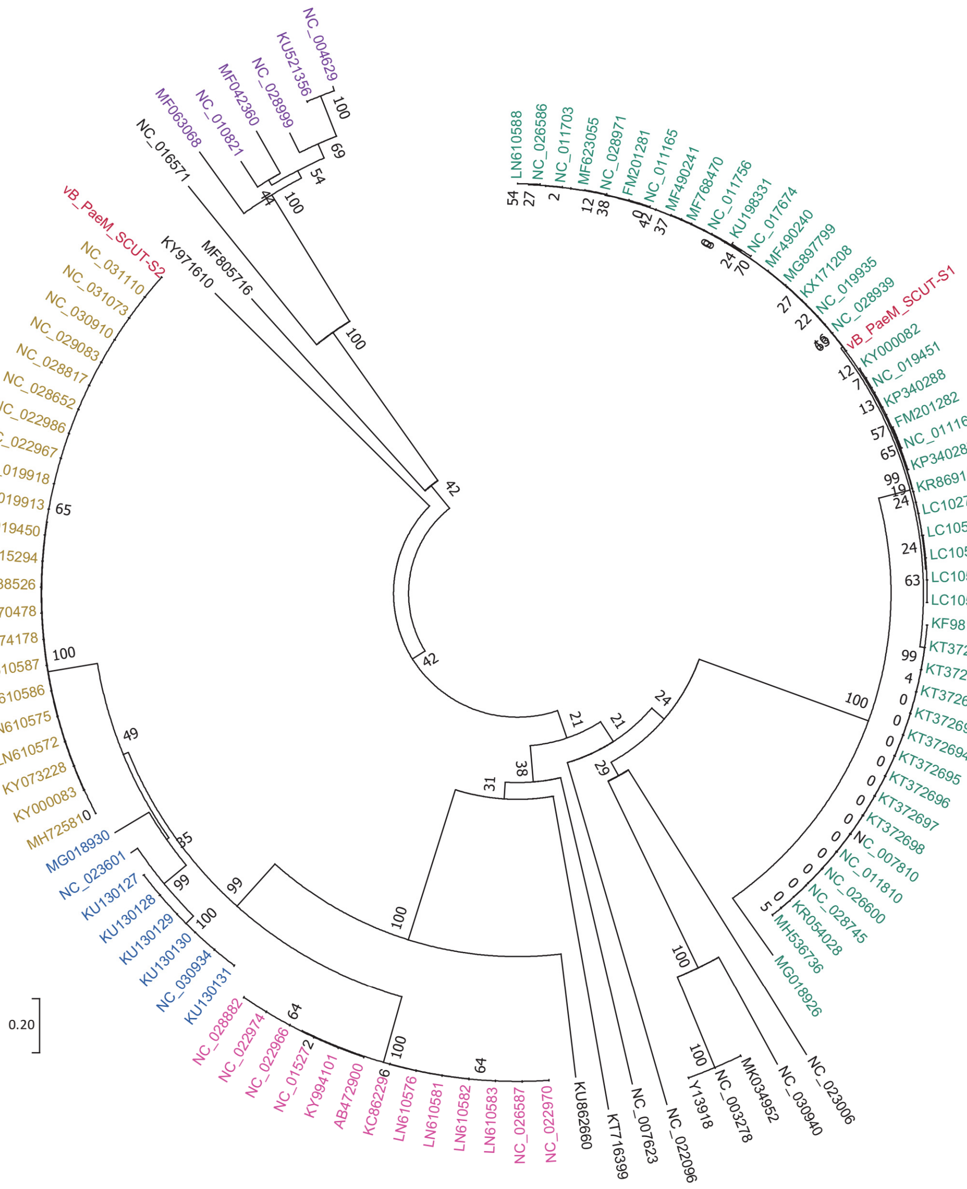

Figure S3. Neighbor-joining tree showing the phylogenetic relationships among Pseudomonas phages of Myoviridae family. The tree was constructed based on the amino acid sequence of the large terminase subunit, the value at the nodes indicated the bootstrap support scores calculated by MEGA 7 using 1000 replicates. The accession number of the complete phage genome were shown.

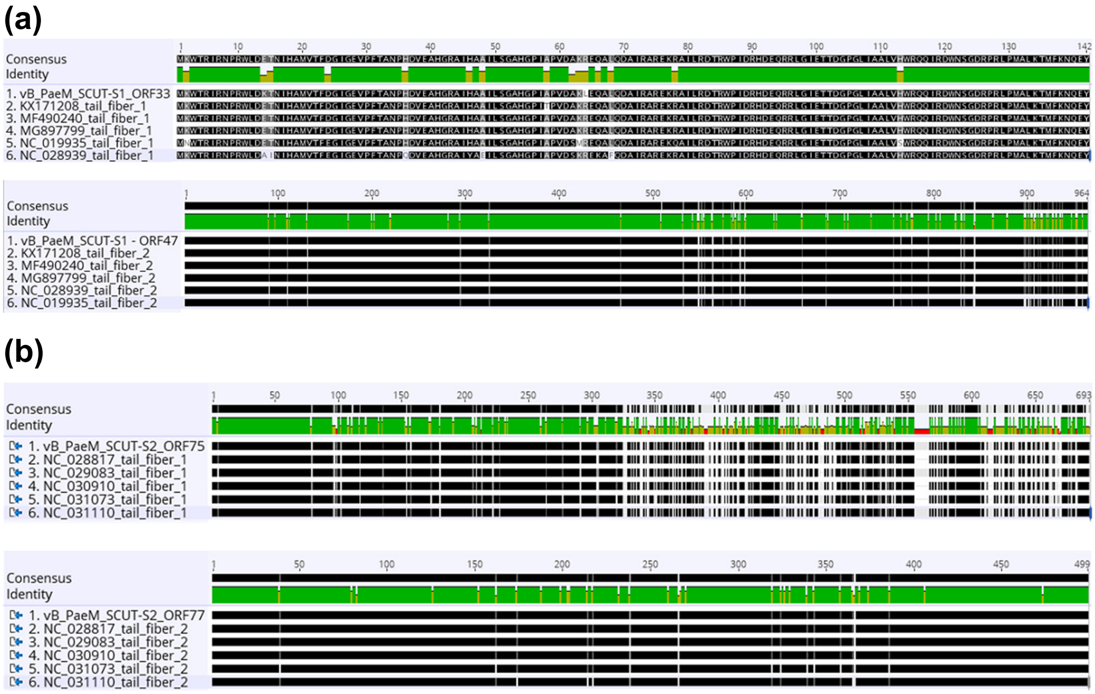

Figure S4. The comparison of the tail fiber proteins of phage vB_PaeM_SCUT-S1 (a) and vB_PaeM_SCUT-S2 (b) to the five most closely related phages.

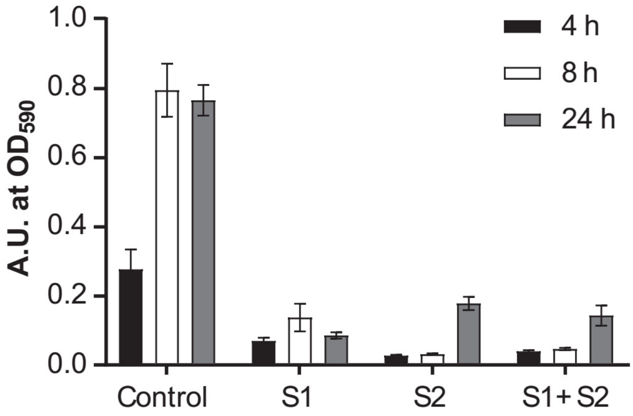

Figure S5. The inhibitory effect of phage treatment on biofilm formation based on the biomass tested by CV staining; Control, without any phages; S1, treated with 10^8 pfu of phage vB_PaeM_SCUT-S1; S2, treated with 10^8 pfu phage vB_PaeM_SCUT-S2; S1+S2, treated with a mixture of vB_PaeM_SCUT-S1 and vB_PaeM_SCUT-S2 (0.5×10^8 pfu of each phage). The different treatment duration is indicated by different bar colors.

Table S1. Host range of phage vB_PaeM_SCUT-S1 and vB_PaeM_SCUT-S2.

Species	Strains	vB_PaeM_SCUT-S1	vB_PaeM_SCUT-S2
Pseudomonas aeruginosa	ATCC 9027	-	T C
	ATCC 15442	C++	+++ C
	ATCC 27853	C+	++ C+
	PAO1	C+++	++ C+
	PALWL1.001	C+++	++ C+
	PALWL1.002	C+++	++ C+
	PALWL1.003	C++	+
Stenotrophomonas maltophilia	ATCC 51331	-	-

"C+++", a large lysis zone at 10^2 pfu; "C++", individual plaques at 10^2 -10^3 pfu; "C+", individual plaques at 10^4 -10^5 pfu; T, turbid lysis zone at 10^6 -10^7 pfu; -, no lysis.

Table S2. The genome annotation of *Pseudomonas* phage vB_PaeM_SCUT-S1

Gene	Start	Stop	Strand	Product length (AA)	Putative function	Query cover(%)	Identity (%)	E-value	Source	GenBank accession No.
1	303	1	-	100	hypothetical protein	98.00	100.00	3.55E-66	phage JG024	YP_006200766.1
2	461	315	-	48	hypothetical protein	100.00	100.00	6.96E-27	phage NH-4	YP_007002548.1
3	832	659	-	57	hypothetical protein	100.00	93.00	1.75E-33	phage vB_PaeM_PAO1_Ab27	YP_009124324.1
4	1165	878	-	95	hypothetical protein	100.00	100.00	3.75E-41	phage NH-4	YP_007002549.1
5	1240	2622	+	460	putative terminase, large subunit	99.78	100.00	0	phage KPP12	YP_007238156.1
6	3042	2659	-	127	hypothetical protein	99.21	100.00	3.12E-88	phage KPP12	YP_007238158.1
7	3257	3039	-	72	hypothetical protein	100.00	100.00	6.41E-49	phage NH-4	YP_007002552.1
8	3589	3257	-	110	hypothetical protein	100.00	100.00	4.79E-79	phage NH-4	YP_007002553.1
9	4031	3633	-	132	hypothetical protein	99.24	100.00	1.81E-91	phage NH-4	YP_007002554.1
10	4813	4034	-	259	hypothetical protein	98.84	100.00	0	phage LBL3	YP_002154152.1
11	5337	4900	-	145	hypothetical protein	86.21	100.00	5.28E-89	phage DL60	YP_009193732.1
12	5942	5355	-	195	hypothetical protein	100.00	100.00	9.54E-148	phage JG024	YP_006200776.1
13	6047	5952	-	31	hypothetical protein	96.77	100.00	2.76E-15	phage KPP12	YP_007238165.1
14	6976	6044	-	310	hypothetical protein	99.36	100.00	0	phage NH-4	YP_007002559.1
15	7427	7080	-	115	hypothetical protein	98.26	100.00	1.39E-78	phage LBL3	YP_002154156.1
16	7987	7676	-	103	hypothetical protein	100.00	100.00	9.39E-71	phage vB_PaeM_PAO1_Ab27	YP_009124336.1
17	8196	7993	-	67	hypothetical protein	97.02	100.00	5.6E-42	phage PB1	YP_002455945.1
18	8516	8193	-	107	hypothetical protein	100.00	100.00	8.97E-78	phage PB1	YP_002455946.1
19	8949	8548	-	133	hypothetical protein	98.50	100.00	2.78E-93	phage F8	YP_001294433.1
20	9129	11426	+	765	putative minor head protein	99.87	100.00	0	phage F8	YP_001294434.1
21	11426	12262	+	278	putative minor head protein	99.64	100.00	0	phage F8	YP_001294435.1
22	12282	12488	+	68	hypothetical protein	100.00	100.00	7.26E-44	phage F8	YP_001294436.1
23	12485	12622	+	45	hypothetical protein	100.00	100.00	3.6E-25	phage F8	YP_001294437.1
24	13138	14574	+	478	putative structural protein	100.00	100.00	0	phage F8	YP_001294438.1
25	14578	15213	+	211	putative structural protein	99.53	100.00	9.2E-150	phage F8	YP_001294439.1
26	15223	16371	+	382	putative major structural protein	99.74	100.00	0	phage F8	YP_001294440.1
27	16473	16910	+	145	hypothetical protein	100.00	100.00	2.67E-107	phage F8	YP_001294441.1
28	16925	17392	+	155	putative structural protein	98.71	100.00	8.64E-111	phage F8	YP_001294442.1
29	17389	17787	+	132	putative structural protein	95.46	100.00	2.49E-90	phage DL60	YP_009193804.1
30	17795	18346	+	183	putative structural protein	97.81	100.00	1.85E-134	phage F8	YP_001294444.1
31	18343	18924	+	193	hypothetical protein	100.00	100.00	3.34E-140	phage NH-4	YP_007002576.1
32	18941	20455	+	504	putative structural protein	99.60	100.00	0	phage F8	YP_001294446.1
33	20513	20965	+	150	putative tail fiber protein	100.00	100.00	7.79E-106	phage JG024	YP_006200796.1
34	20965	21288	+	107	putative structural protein	100.00	100.00	4.34E-78	phage PB1	YP_002455962.1
35	21285	21635	+	116	putative structural protein	100.00	100.00	3.3E-81	phage LBL3	YP_002154175.1
36	21637	22065	+	142	putative structural protein	94.37	100.00	5.36E-91	phage DL60	YP_009193798.1
37	22075	22578	+	167	putative structural protein	100.00	100.00	3.78E-118	phage PB1	YP_002455965.1
38	22578	23117	+	179	putative structural protein	100.00	100.00	8.88E-131	phage PB1	YP_002455966.1
39	23126	23719	+	197	putative tail fiber protein	98.99	100.00	9.97E-142	phage F8	YP_001294453.1
40	23729	24157	+	142	hypothetical protein	100.00	100.00	8.36E-104	phage PB1	YP_002455968.1
41	24161	26743	+	860	putative tail protein containing transglycosylase	99.77	100.00	0	phage F8	YP_001294455.1
42	26743	27606	+	287	putative structural protein	99.65	100.00	0	phage F8	YP_001294456.1
43	27606	28139	+	177	hypothetical protein	100.00	100.00	4.35E-131	phage LBL3	YP_002154183.1
44	28195	28860	+	221	putative baseplate protein	100.00	100.00	3.32E-164	phage LBL3	YP_002154184.1
45	28917	30170	+	417	putative baseplate protein	99.04	100.00	0	phage DL68	YP_009215185.1
46	30167	31681	+	504	putative structural protein	99.01	100.00	0	phage KPP12	YP_007238197.1
47	31686	34580	+	964	putative tail fiber protein	99.07	100.00	0	phage vB_PaeM_PAO1_Ab27	YP_009124366.1
48	34582	35010	+	142	putative tail fiber protein	97.89	100.00	1.51E-100	phage vB_PaeM_PAO1_Ab27	YP_009124367.1
49	35010	35672	+	220	putative endolysin	100.00	100.00	1.45E-165	phage KPP12	YP_007238200.1
50	35948	35697	-	83	hypothetical protein	100.00	100.00	2.41E-53	phage JG024	YP_006200813.1
51	37139	36228	-	303	putative DNA ligase	99.34	100.00	0	phage LMA2	YP_002154282.1
52	37748	37194	-	184	putative DNA-binding protein	99.46	100.00	2.57E-135	phage NH-4	YP_007002597.1
53	38350	37745	-	201	putative holin	100.00	100.00	5.33E-142	phage KPP12	YP_007238204.1
54	39303	38404	-	299	hypothetical protein	99.67	100.00	0	phage 14-1	YP_002364360.1
55	40012	39392	-	206	hypothetical protein	99.52	100.00	1.86E-147	phage SN	YP_002418860.1
56	41666	40107	-	519	putative DNA helicase	100.00	100.00	0	phage KPP12	YP_007238207.1
57	42073	41663	-	136	putative DNA helicase	100.00	100.00	8.77E-98	phage NH-4	YP_007002602.1
58	45173	42066	-	1035	putative DNA polymerase III alpha subunit	99.81	100.00	0	phage KPP12	YP_007238209.1
59	45727	45173	-	184	putative DNA polymerase III epsilon subunit	100.00	100.00	2.53E-138	phage NH-4	YP_007002604.1
60	46819	45803	-	338	putative polynucleotide kinase	100.00	100.00	0	phage KPP12	YP_007238211.1
61	47013	46822	-	63	hypothetical protein	100.00	100.00	2.83E-39	phage JG024	YP_006200824.1

62	47932	47015	-	305	putative thymidylate synthase	100.00	100.00	0	phage SN	YP_002418867.1
63	48138	47932	-	68	hypothetical protein	94.12	100.00	1.67E-41	phage NH-4	YP_007002608.1
64	48373	48146	-	75	hypothetical protein	100.00	100.00	2.65E-51	phage KPP12	YP_007238215.1
65	48624	48406	-	72	putative tail assembly protein	100.00	100.00	2.69E-49	phage KPP12	YP_007238216.1
66	48826	48608	-	72	hypothetical protein	100.00	100.00	3.71E-46	phage KPP12	YP_007238217.1
67	49056	48826	-	76	hypothetical protein	98.68	100.00	5.3E-52	phage KPP12	YP_007238218.1
68	50145	49144	-	333	hypothetical protein	99.40	100.00	0	phage KPP12	YP_007238219.1
69	51143	50250	-	297	putative structural protein	98.65	100.00	0	phage F8	YP_001294483.1
70	52491	51304	-	395	putative ATP-dependent exonuclease V	100.00	100.00	0	phage F8	YP_001294484.1
71	52900	52478	-	140	hypothetical protein	100.00	100.00	3.22E-101	phage LMA2	YP_002154302.1
72	53069	53854	+	261	hypothetical protein	98.85	100.00	0	phage LMA2	YP_002154303.1
73	53865	54839	+	324	hypothetical protein	61.17	100.00	1.01E-137	phage LMA2	YP_002154304.1
74	54863	55321	+	152	hypothetical protein	100.00	100.00	2.57E-110	phage LMA2	YP_002154305.1
75	55318	56394	+	358	hypothetical protein	100.00	100.00	0	phage KPP12	YP_007238226.1
76	56400	56585	+	61	hypothetical protein	100.00	100.00	1.2E-36	phage JG024	YP_006200840.1
77	56817	58472	+	551	putative DNA primase	99.27	100.00	0	phage LMA2	YP_002154308.1
78	58670	59299	+	209	hypothetical protein	74.65	100.00	3.61E-103	phage KPP12	YP_007238229.1
79	59479	60048	+	189	hypothetical protein	99.47	100.00	1.32E-140	phage JG024	YP_006200844.1
80	60832	60218	-	204	hypothetical protein	99.51	100.00	8.2E-147	phage LMA2	YP_002154310.1
81	61740	61021	-	239	hypothetical protein	88.24	99.00	5.01E-134	phage vB_PaeM_PAO1_Ab27	YP_009124306.1
82	62062	61751	-	103	hypothetical protein	97.09	100.00	5.08E-74	phage SN	YP_002418886.1
83	62345	62115	-	76	hypothetical protein	96.05	100.00	9.05E-45	phage PB1	YP_002456012.1
84	62625	62401	-	74	hypothetical protein	98.65	100.00	7.94E-50	phage NH-4	YP_007002630.1
85	63016	62690	-	108	hypothetical protein	98.15	100.00	1.22E-75	phage NH-4	YP_007002631.1
86	63661	63017	-	214	hypothetical protein	99.07	100.00	1.79E-157	phage NH-4	YP_007002632.1
87	63905	63693	-	70	hypothetical protein	95.71	100.00	2.75E-47	phage vB_PaeM_PAO1_Ab27	YP_009124313.1
88	64102	63902	-	66	hypothetical protein	100.00	100.00	2.23E-43	phage NH-4	YP_007002634.1
89	64314	64099	-	71	hypothetical protein	100.00	100.00	4.63E-47	phage NH-4	YP_007002635.1
90	64511	64311	-	66	hypothetical protein	98.49	100.00	7E-43	phage NH-4	YP_007002636.1
91	64759	64508	-	83	hypothetical protein	98.80	100.00	5.66E-57	phage NH-4	YP_007002637.1
92	65070	64885	-	61	hypothetical protein	100.00	98.00	1.04E-37	phage LMA2	YP_002154323.1
93	65343	65155	-	62	hypothetical protein	91.94	100.00	2.95E-39	phage LMA2	YP_002154324.1
94	66032	65346	-	228	putative tail length tape-measure protein	86.78	99.00	0	phage vB_Pae_PS44	YP_009211418.1

Table S3. The genome annotation of *Pseudomonas* phage vB_PaeM_SCUT-S2

Gene	Start	Stop	Strand	Product length (AA)	Putative function	Query cover(%)	Identity (%)	E-value	Source	GenBank accession No.
1	55	204	+	49	hypothetical protein	-	-	-	-	-
2	344	946	+	200	hypothetical protein	99.00	100.00	5.83E-153	phage K5	YP 009273933.1
3	1468	1626	+	52	hypothetical protein	100.00	100.00	2.02E-31	phage JG004	YP 007002525.1
4	1946	1680	-	88	hypothetical protein	100.00	100.00	7.68E-63	phage PAK P4	YP 008859337.1
5	2349	1939	-	136	hypothetical protein	99.27	100.00	2.19E-99	phage PAK P4	YP 008859338.1
6	2755	2339	-	138	hypothetical protein	99.28	100.00	8.76E-101	phage PAK P4	YP 008859339.1
7	3484	2810	-	224	hypothetical protein	98.66	100.00	2.23E-162	phage PAK P2	YP 008857170.1
8	3765	3487	-	92	hypothetical protein	100.00	100.00	2.09E-60	phage PAK P4	YP 008859341.1
9	4064	3753	-	103	hypothetical protein	100.00	100.00	1.94E-70	phage JG004	YP 007002519.1
10	4426	4064	-	120	hypothetical protein	100.00	100.00	6E-87	phage PAK P4	YP 008859343.1
11	4698	4483	-	71	hypothetical protein	100.00	100.00	3.88E-47	phage K5	YP 009273767.1
12	5036	4695	-	113	hypothetical protein	97.35	100.00	1.08E-79	phage PaoP5	YP 009224704.1
13	5541	5038	-	167	hypothetical protein	98.20	100.00	9.6E-123	phage PaoP5	YP 009224705.1
14	5905	5528	-	125	hypothetical protein	100.00	100.00	1.06E-91	phage phiMK	YP 009291226.1
15	6506	5925	-	193	hypothetical protein	99.48	100.00	6.42E-143	phage PAK P1	YP 004327157.1
16	6820	6503	-	105	hypothetical protein	98.10	100.00	3.05E-74	phage PAK P1	YP 004327158.1
17	6971	6822	-	49	hypothetical protein	100.00	100.00	3.41E-29	phage vB PaeM MAG1	YP_009287453.1
18	7462	6983	-	159	hypothetical protein	100.00	100.00	2.8E-117	phage K5	YP 009273774.1
19	7656	7459	-	65	hypothetical protein	100.00	100.00	1.49E-44	phage K5	YP 009273775.1
20	9356	7668	-	562	putative nictotinate phosphoribosyltransferase	99.82	100.00	0	phage K5	YP_009273776.1
21	9631	9413	-	72	hypothetical protein	100.00	100.00	9.6E-47	phage K5	YP 009273778.1
22	10494	9628	-	288	putative phosphoribosylpyrophosphate synthetase	100.00	100.00	0	phage K5	YP_009273779.1
23	10920	10504	-	138	putative ATPase	100.00	100.00	7.59E-101	phage K5	YP 009273780.1
24	11848	10931	-	305	putative RNA ligase/tail attachment protein	100.00	100.00	0	phage C11	YP_009186914.1
25	12267	11860	-	135	hypothetical protein	100.00	100.00	2.54E-94	phage vB_PaeM_C2-10 Ab1	YP_007236843.1
26	12539	12264	-	91	putative helix-turn-helix motif	100.00	100.00	1.89E-64	phage vB_PaeM_C2-10 Ab1	YP_007236844.1
27	12777	12541	-	78	hypothetical protein	100.00	100.00	5.48E-52	phage JG004	YP 007002503.1
28	13344	12790	-	184	putative phosphoesterase	99.46	100.00	6.8E-137	phage JG004	YP 007002502.1
29	13775	13344	-	143	hypothetical protein	95.11	100.00	5.1E-99	phage PaoP5	YP 009224722.1
30	14319	13765	-	184	putative phosphohydrolase	93.55	100.00	2.7E-130	phage PaoP5	YP_009224723.1
31	14881	14321	-	186	putative cell wall hydrolase	100.00	100.00	5.18E-141	phage vB_PaeM_C2-10 Ab1	YP_007236848.1
32	15407	14946	-	153	hypothetical protein	96.08	100.00	8.49E-110	phage vB_PaeM_C2-10 Ab1	YP_007236850.1
33	16628	15420	-	402	putative DNA ligase	99.25	100.00	0	phage PAK P4	YP 008859367.1
34	17041	16625	-	138	putative dCMP deaminase	100.00	100.00	1.01E-99	phage JG004	YP 007002496.1
35	17271	17044	-	75	hypothetical protein	100.00	100.00	3.83E-50	phage PAK P4	YP 008859369.1
36	17493	17281	-	70	hypothetical protein	100.00	100.00	1.67E-45	phage PAK P4	YP 008859370.1
37	17759	17490	-	89	hypothetical protein	100.00	100.00	2.57E-61	phage vB_PaeM_C2-10 Ab1	YP_007236855.1
38	18118	17768	-	116	hypothetical protein	100.00	100.00	2.16E-83	phage vB_PaeM_C2-10 Ab1	YP_007236856.1
39	18377	18099	-	92	hypothetical protein	100.00	100.00	2.46E-63	phage C11	YP 009186929.1
40	18652	18374	-	92	hypothetical protein	100.00	100.00	1.05E-64	phage C11	YP 009186930.1
41	18872	18687	-	61	hypothetical protein	100.00	100.00	3.08E-41	phage C11	YP 009186931.1
42	19115	18873	-	80	hypothetical protein	100.00	100.00	1.04E-54	phage K5	YP 009273799.1
43	19297	19112	-	61	hypothetical protein	98.36	100.00	8.67E-38	phage K5	YP 009273800.1
44	19906	19358	-	182	putative protease subunit	99.45	100.00	7.71E-135	phage vB_PaeM_C2-10 Ab1	YP_007236861.1
45	20310	19954	-	118	hypothetical protein	100.00	100.00	9.21E-81	phage vB_PaeM_C2-10 Ab1	YP_007236862.1
46	20774	20307	-	155	hypothetical protein	100.00	100.00	2.63E-113	phage JG004	YP 007002484.1
47	21249	21437	+	62	hypothetical protein	100.00	100.00	3.07E-42	phage vB_PaeM_C2-10 Ab1	YP_007236865.1
48	21580	21903	+	107	hypothetical protein	100.00	100.00	4.03E-74	phage PAK P1	YP 004327192.1
49	22070	22264	+	64	hypothetical protein	100.00	100.00	2.44E-41	phage PAK P1	YP 004327193.1
50	23341	23210	-	43	hypothetical protein	100.00	100.00	2.06E-25	phage PAK P2	YP 008857216.1
51	25276	26796	+	506	putative terminase large subunit	100.00	100.00	0	phage vB_PaeM_C2-10 Ab1	YP_007236868.1

52	26809	28248	+	479	putative structural protein	100.00	100.00	0	phage vB_PaeM_C2-10_Ab1	YP_007236869.1
53	28258	28728	+	156	putative methyltransferase	100.00	100.00	7.05E-113	phage vB_PaeM_C2-10_Ab1	YP_007236870.1
54	28725	29642	+	305	hypothetical protein	100.00	100.00	0	phage vB_PaeM_C2-10_Ab1	YP_007236871.1
55	29670	30080	+	136	putative structural protein	99.27	100.00	6.46E-97	phage K5	YP_009273810.1
56	30124	31158	+	344	Major capsid protein	100.00	100.00	0	phage vB_PaeM_C2-10_Ab1	YP_007236873.1
57	31209	31685	+	158	hypothetical protein	100.00	100.00	5.8E-116	phage vB_PaeM_C2-10_Ab1	YP_007236874.1
58	31723	32136	+	137	putative RNA polymerase	100.00	100.00	1.51E-101	phage PAK_P4	YP_008859219.1
59	32136	32516	+	126	hypothetical protein	100.00	100.00	8.84E-93	phage JG004	YP_007002473.1
60	32513	33076	+	187	putative structural protein	100.00	100.00	7.34E-142	phage vB_PaeM_C2-10_Ab1	YP_007236877.1
61	33089	34375	+	428	putative structural protein	99.77	100.00	0	phage vB_PaeM_MAG1	YP_009287410.1
62	34406	34930	+	174	putative structural protein	100.00	100.00	1.3E-127	phage vB_PaeM_C2-10_Ab1	YP_007236879.1
63	35005	35505	+	166	hypothetical protein	100.00	100.00	6.14E-122	phage vB_PaeM_C2-10_Ab1	YP_007236880.1
64	35505	35984	+	159	putative structural protein	100.00	100.00	5.25E-116	phage vB_PaeM_C2-10_Ab1	YP_007236881.1
65	35998	36369	+	123	putative structural protein	99.19	100.00	3.52E-87	phage vB_PaeM_C2-10_Ab1	YP_007236882.1
66	36477	36629	+	50	hypothetical protein	100.00	100.00	1.71E-29	phage PAK_P4	YP_008859228.1
67	36626	38992	+	788	putative tape measure protein	100.00	100.00	0	phage vB_PaeM_C2-10_Ab1	YP_007236883.1
68	38989	39750	+	253	hypothetical protein	98.81	100.00	0	phage vB_PaeM_C2-10_Ab1	YP_007236884.1
69	39756	40112	+	118	hypothetical protein	100.00	100.00	5.03E-85	phage vB_PaeM_C2-10_Ab1	YP_007236886.1
70	40109	41026	+	305	hypothetical protein	100.00	100.00	0	phage PAK_P4	YP_008859232.1
71	41023	41763	+	246	putative baseplate protein	100.00	100.00	0	phage vB_PaeM_C2-10_Ab1	YP_007236888.1
72	41774	42145	+	123	hypothetical protein	100.00	100.00	2.91E-88	phage vB_PaeM_C2-10_Ab1	YP_007236889.1
73	42147	43610	+	487	putative baseplate component	100.00	100.00	0	phage vB_PaeM_C2-10_Ab1	YP_007236890.1
74	43629	44360	+	243	hypothetical protein	100.00	100.00	3.39E-177	phage vB_PaeM_C2-10_Ab1	YP_007236891.1
75	44371	46428	+	685	putative tail fiber protein	97.37	99.00	0	phage PaoP5	YP_009224767.1
76	46472	46846	+	124	putative tail fiber assembly protein	100.00	100.00	6.82E-89	phage phiMK	YP_009291165.1
77	46860	48359	+	499	putative tail fiber protein	99.80	100.00	0	phage K5	YP_009273832.1
78	48376	48936	+	186	putative endolysin	100.00	100.00	7.06E-140	phage PaoP5	YP_009224770.1
79	48954	49193	+	79	hypothetical protein	100.00	100.00	1.82E-51	phage PAK_P4	YP_008859241.1
80	49180	49617	+	145	hypothetical protein	100.00	100.00	4.73E-106	phage PaoP5	YP_009224772.1
81	49755	50060	+	101	hypothetical protein	100.00	100.00	3.15E-68	phage vB_PaeM_C2-10_Ab1	YP_007236898.1
82	50096	50410	+	104	hypothetical protein	100.00	100.00	1.38E-71	phage phiMK	YP_009291158.1
83	50759	50448	-	103	hypothetical protein	100.00	100.00	2.13E-71	phage PaoP5	YP_009224775.1
84	51091	50771	-	106	hypothetical protein	98.11	100.00	9.49E-75	phage phiMK	YP_009291156.1
85	51918	51109	-	269	putative pyrophosphatase	97.03	100.00	0	phage vB_PaeM_C2-10_Ab1	YP_007236901.1
86	52069	51911	-	52	hypothetical protein	100.00	100.00	4.75E-31	phage C11	YP_009186975.1
87	53227	52082	-	381	Putative RNA ligase	98.16	100.00	0	phage phiMK	YP_009291153.1
88	53492	53259	-	77	hypothetical protein	100.00	100.00	1.03E-51	phage JG004	YP_007002443.1
89	53763	53530	-	77	hypothetical protein	100.00	100.00	6.89E-51	phage JG004	YP_007002442.1
90	54218	54415	+	65	hypothetical protein	100.00	100.00	1.62E-42	phage vB_PaeM_C2-10_Ab1	YP_007236905.1
91	54418	54903	+	161	hypothetical protein	88.20	100.00	6.78E-108	phage PAK_P1	YP_008869165.1

92	54936	55190	+	84	hypothetical protein	100.00	100.00	1.68E-57	phage vB_PaeM_C2-10_Ab1	YP_007236908.1
93	55192	55581	+	129	hypothetical protein	98.45	100.00	7.54E-93	phage vB_PaeM_C2-10_Ab1	YP_007236909.1
94	55578	56243	+	221	hypothetical protein	100.00	100.00	6.13E-164	phage PAK_P1	YP_004327236.1
95	56230	56394	+	54	hypothetical protein	100.00	100.00	2.28E-34	phage vB_PaeM_C2-10_Ab1	YP_007236911.1
96	56397	56699	+	100	hypothetical protein	100.00	100.00	1.75E-70	phage K5	YP_009273851.1
97	56700	57122	+	140	hypothetical protein	100.00	100.00	3.29E-105	phage PAK_P4	YP_008859259.1
98	57131	57247	+	38	hypothetical protein	97.37	100.00	6.96E-22	phage PAK_P4	YP_008859260.1
99	57240	57368	+	42	hypothetical protein	100.00	100.00	6.51E-24	phage phiMK	YP_009291143.1
100	57355	57546	+	63	putative RNA methyltransferase	100.00	100.00	3.43E-40	phage phiMK	YP_009291142.1
101	57556	57801	+	81	putative DNA primase/helicase	98.77	100.00	7.04E-57	phage vB_PaeM_C2-10_Ab1	YP_007236915.1
102	57798	57983	+	61	hypothetical protein	100.00	100.00	1.32E-41	phage JG004	YP_007002431.1
103	58037	59899	+	620	hypothetical protein	100.00	100.00	0	phage JG004	YP_007003114.1
104	59960	62674	+	904	putative DNA polymerase	99.34	100.00	0	phage C11	YP_009186992.1
105	62764	63162	+	132	hypothetical protein	100.00	100.00	4.84E-92	phage PAK_P4	YP_008859267.1
106	63185	63352	+	55	hypothetical protein	100.00	100.00	9.67E-35	phage vB_PaeM_C2-10_Ab1	YP_007236920.1
107	63354	64070	+	238	hypothetical protein	100.00	100.00	4.34E-178	phage JG004	YP_007002427.1
108	64172	65176	+	334	hypothetical protein	99.40	100.00	0	phage JG004	YP_007002426.1
109	65246	65479	+	77	hypothetical protein	100.00	100.00	4.64E-48	phage JG004	YP_007002425.1
110	65489	65710	+	73	hypothetical protein	100.00	100.00	6.35E-50	phage JG004	YP_007002424.1
111	65752	66804	+	350	putative exodeoxyribonuclease	99.71	100.00	0	phage C11	YP_009186999.1
112	66801	67364	+	187	hypothetical protein	100.00	100.00	2.44E-138	phage vB_PaeM_MAG1	YP_009287360.1
113	67361	67759	+	132	hypothetical protein	100.00	100.00	2.25E-96	phage C11	YP_009187001.1
114	67756	67992	+	78	hypothetical protein	100.00	100.00	9.28E-52	phage PAK_P1	YP_008869173.1
115	67989	68426	+	145	hypothetical protein	100.00	100.00	5.53E-105	phage C11	YP_009187003.1
116	68423	68590	+	55	hypothetical protein	100.00	100.00	4.1E-35	phage K5	YP_009273872.1
117	68592	68774	+	60	hypothetical protein	100.00	100.00	2.07E-38	phage K5	YP_009273873.1
118	68771	69550	+	259	hypothetical protein	100.00	100.00	0	phage PAK_P4	YP_008859280.1
119	69547	69729	+	60	hypothetical protein	100.00	100.00	2.58E-38	phage vB_PaeM_MAG1	YP_009287353.1
120	69741	69950	+	69	hypothetical protein	100.00	100.00	1.91E-44	phage vB_PaeM_MAG1	YP_009287352.1
121	69969	70304	+	111	hypothetical protein	100.00	100.00	4.7E-77	phage vB_PaeM_C2-10_Ab1	YP_007236933.1
122	70308	70523	+	71	hypothetical protein	100.00	100.00	1.81E-45	phage vB_PaeM_MAG1	YP_009287350.1
123	70516	71466	+	316	putative 3'-phosphatase	98.73	100.00	0	phage JG004	YP_007002411.1
124	71502	71618	+	38	hypothetical protein	97.37	100.00	4.49E-21	phage PAK_P4	YP_008859286.1
125	71673	72620	+	315	Putative thymidylate synthase	99.68	100.00	0	phage JG004	YP_007002410.1
126	72623	72967	+	114	hypothetical protein	100.00	100.00	1.75E-83	phage PaP1	YP_007236522.1
127	72984	74030	+	348	putative ribonucleoside-diphosphate reductase beta subunit	100.00	100.00	0	phage JG004	YP_007002408.1
128	74023	75768	+	581	putative ribonucleoside-diphosphate reductase alpha chain	99.83	100.00	0	phage C11	YP_009187016.1
129	75843	75989	+	48	hypothetical protein	100.00	100.00	2.28E-30	phage PAK_P4	YP_008859291.1
130	75986	76213	+	75	hypothetical protein	100.00	100.00	4.59E-51	phage PAK_P4	YP_008859292.1
131	76213	76446	+	77	hypothetical protein	100.00	100.00	4.95E-54	phage JG004	YP_007002405.1
132	76446	76682	+	78	hypothetical protein	100.00	100.00	1.16E-52	phage vB_PaeM_C2-10_Ab1	YP_007236942.1
133	76682	76996	+	104	hypothetical protein	100.00	100.00	2.13E-74	phage vB_PaeM_C2-10_Ab1	YP_007236943.1
134	76986	77171	+	61	hypothetical protein	100.00	100.00	2.56E-40	phage vB_PaeM_C2-10_Ab1	YP_007236944.1
135	77212	77460	+	82	hypothetical protein	100.00	100.00	1.52E-55	phage PAK_P2	YP_008857125.1
136	77472	77957	+	161	hypothetical protein	100.00	100.00	2.74E-117	phage C11	YP_009187024.1
137	77967	78161	+	64	hypothetical protein	100.00	100.00	6.9E-42	phage vB_PaeM_C2-10_Ab1	YP_007236947.1
138	78163	78402	+	79	hypothetical protein	100.00	100.00	4.21E-54	phage vB_PaeM_C2-10_Ab1	YP_007236948.1
139	78465	78665	+	66	hypothetical protein	100.00	100.00	3.1E-43	phage vB_PaeM_C2-10_Ab1	YP_007236949.1

140	78833	79819	+	328	hypothetical protein	100.00	100.00	0	phage C11	YP 009187028.1
141	80039	80212	+	57	hypothetical protein	100.00	100.00	1.7E-36	phage K8	YP 009200080.1
142	80841	81317	+	158	hypothetical protein	99.37	100.00	5.36E-117	phage C11	YP 009187030.1
143	81392	81661	+	89	hypothetical protein	100.00	100.00	2.88E-63	phage C11	YP 009187031.1
144	81673	81816	+	47	hypothetical protein	100.00	100.00	5.32E-30	phage C11	YP 009187032.1
145	81816	82106	+	96	hypothetical protein	100.00	100.00	2.86E-66	phage C11	YP 009187033.1
146	82096	82329	+	77	hypothetical protein	100.00	100.00	7.58E-52	phage C11	YP 009187034.1
147	82400	82528	+	42	hypothetical protein	100.00	100.00	8.03E-24	phage JG004	YP 007002390.1
148	82528	82836	+	102	hypothetical protein	100.00	100.00	5.02E-72	phage vB_PaeM_C2-10 Ab1	YP_007236957.1
149	82911	83057	+	48	hypothetical protein	100.00	100.00	6.84E-26	phage C11	YP 009187037.1
150	83152	83301	+	49	hypothetical protein	100.00	100.00	1.31E-31	phage phiMK	YP 009291091.1
151	83294	83677	+	127	hypothetical protein	100.00	100.00	4.44E-91	phage C11	YP 009187039.1
152	83753	84424	+	223	hypothetical protein	99.10	100.00	2.2E-168	phage vB_PaeM_C2-10 Ab1	YP_007236959.1
153	84429	84767	+	112	hypothetical protein	100.00	100.00	9.09E-83	phage phiMK	YP 009291088.1
154	84767	85120	+	117	hypothetical protein	100.00	100.00	9.36E-84	phage C11	YP 009187042.1
155	85198	85311	+	37	hypothetical protein	81.08	100.00	1.09E-16	phage PAK P4	YP 008859315.1
156	85384	85578	+	64	hypothetical protein	100.00	100.00	8.32E-42	phage C11	YP 009187043.1
157	85594	85890	+	98	hypothetical protein	100.00	100.00	6.48E-69	phage C11	YP 009187044.1
158	85887	86111	+	74	hypothetical protein	100.00	100.00	8.36E-51	phage C11	YP 009187045.1
159	86144	86410	+	88	hypothetical protein	100.00	100.00	2.82E-61	phage C11	YP 009187046.1
160	86407	86799	+	130	hypothetical protein	100.00	100.00	9.74E-95	phage C11	YP 009187047.1
161	86904	87158	+	84	hypothetical protein	100.00	100.00	2.64E-57	phage K5	YP 009273918.1
162	87236	87592	+	118	hypothetical protein	100.00	100.00	2.99E-83	phage K5	YP 009273919.1
163	87635	88216	+	193	hypothetical protein	99.48	100.00	1.5E-141	phage K5	YP 009273920.1
164	88219	88542	+	107	hypothetical protein	99.07	100.00	2.8E-75	phage C11	YP 009187051.1
165	88578	89057	+	159	hypothetical protein	99.37	100.00	6.74E-117	phage C11	YP 009187052.1
166	89143	89670	+	175	hypothetical protein	100.00	100.00	1.31E-128	phage PaP1	YP 007236558.1
167	89670	89951	+	93	hypothetical protein	100.00	100.00	3.13E-66	phage PaP1	YP 007236559.1
168	89978	90142	+	54	hypothetical protein	98.15	100.00	1.98E-33	phage K5	YP 009273926.1
169	90223	90366	+	47	hypothetical protein	97.87	100.00	2.87E-28	phage C11	YP 009187055.1
170	90430	90891	+	153	hypothetical protein	98.04	100.00	1.4E-111	phage phiMK	YP 009291072.1
171	90908	91210	+	100	hypothetical protein	100.00	100.00	2.22E-69	phage phiMK	YP 009291071.1
172	91304	91570	+	88	hypothetical protein	98.86	100.00	3.92E-61	phage vB_PaeM_C2-10 Ab1	YP_007236977.1
173	91670	91864	+	64	hypothetical protein	98.44	100.00	5.81E-41	phage C11	YP 009187059.1
174	91914	92054	+	46	hypothetical protein	-	-	-	-	-
175	92059	92259	+	66	hypothetical protein	76.92	85.00	1.46E-16	phage PaoP5	YP 009224863.1
176	92300	92461	+	53	hypothetical protein	98.11	100.00	7.44E-34	phage PAK P4	YP 008859332.1
177	92516	92776	+	86	hypothetical protein	98.84	100.00	5.47E-56	phage PAK P4	YP 008859333.1
178	93301	93450	+	49	hypothetical protein	-	-	-	-	-
179	93590	94192	+	200	hypothetical protein	99.00	100.00	0	phage K5	YP 009273933.1

Table S4. Amino acid identity between phage vB_PaeM_SCUT-S1 (a) and phage vB_PaeM_SCUT-S2 (b) with five most similar members indicated by BLASTN analysis

(a)

Amino acid identity (100%)	DQ163917.1 (F8)	KP340288.1 (phiKTN6)	KR869157.1 (vB_PaeM_ CEB_DP1)	MF490240.1 (vB_PaeM_E 217)	LN610579.1 (vB_PaeM_P AO1_Ab27)
100	16	10	15	16	12
95-100	37	46	52	50	52
90-95	13	21	14	14	18
80-90	12	6	7	2	6
70-80	7	4	3	5	2
60-70	1	2	1	1	0
50-60	4	2	0	1	1
30-50	1	0	0	2	0
0	3	3	2	3	3

(b)

amino acid identity (100%)	GU988610.1 (JG004)	KT804923.1 (C11)	KY073228.1 (Zigelbrucke)	MH370478.1 (SRT6)	MH725810.1 (PaYy-2)
100	44	79	46	94	107
95-100	71	71	74	51	45
90-95	21	14	17	10	11
80-90	11	6	17	7	6
70-80	7	0	3	2	1
60-70	4	1	4	2	1
50-60	5	1	3	1	1
30-50	5	0	6	2	1
0	11	7	9	10	6
